# Molecular Diagnosis of Hemorrhagic Fever with Renal Syndrome Caused by Puumala Virus

**DOI:** 10.1128/JCM.00113-16

**Published:** 2016-04-25

**Authors:** Nina Lagerqvist, Åsa Hagström, Malin Lundahl, Elin Nilsson, Mikael Juremalm, Inger Larsson, Erik Alm, Göran Bucht, Clas Ahlm, Jonas Klingström

**Affiliations:** a Public Health Agency of Sweden, Solna, Sweden; b Center for Infectious Medicine, Department of Medicine Huddinge, Karolinska Institutet, Stockholm, Sweden; c National Veterinary Institute, Uppsala, Sweden; d Swedish Defence Research Agency, Umeå, Sweden; e Sunderbyn Hospital, Luleå, Sweden; f Department of Clinical Microbiology, Infectious Diseases, Umeå University, Umeå, Sweden

## Abstract

Rodent-borne hantaviruses cause two severe acute diseases: hemorrhagic fever with renal syndrome (HFRS) in Eurasia, and hantavirus pulmonary syndrome (HPS; also called hantavirus cardiopulmonary syndrome [HCPS]) in the Americas. Puumala virus (PUUV) is the most common causative agent of HFRS in Europe. Current routine diagnostic methods are based on serological analyses and can yield inconclusive results. Hantavirus-infected patients are viremic during the early phase of disease; therefore, detection of viral RNA genomes can be a valuable complement to existing serological methods. However, the high genomic sequence diversity of PUUV has hampered the development of molecular diagnostics, and currently no real-time reverse transcription-quantitative (RT)-PCR assay is available for routine diagnosis of HFRS. Here, we present a novel PUUV RT-PCR assay. The assay was validated for routine diagnosis of HFRS on samples collected in Sweden during the winter season from 2013 to 2014. The assay allowed detection of PUUV RNA in 98.7% of confirmed clinical HFRS samples collected within 8 days after symptomatic onset. In summary, this study shows that real-time RT-PCR can be a reliable alternative to serological tests during the early phase of HFRS.

## INTRODUCTION

Hantaviruses (family Bunyaviridae) cause two severe acute zoonotic diseases: hemorrhagic fever with renal syndrome (HFRS) in Eurasia, and hantavirus pulmonary syndrome (HPS; also called hantavirus cardiopulmonary syndrome [HCPS]) in the Americas (1). Puumala virus (PUUV) is the major HFRS-causing hantavirus in Europe (2). The number of diagnosed HFRS patients in Europe is increasing; from 1990 to 1999, the average number of cases per year was below 2,000; from 2000 to 2009, it was above 3,000 cases per year (2). Further, underreporting of PUUV-caused HFRS has been described. For example, three patients with suspected leptospirosis were in retrospect diagnosed with HFRS (3). There is a need for rapid, reliable, and easy-to-use diagnostic methods for HFRS. Currently, routine diagnosis of HFRS is based on detection of virus-specific IgM and/or IgG antibodies (4). However, not all patients show detectable antibody levels (5) and, consequently, not all patients can be diagnosed using serological methods, at early stages of the disease. Hantaviruses establish systemic infections, and patients are viremic when they present with symptoms (4, 6–8), indicating that detection of hantavirus RNA has diagnostic potential. PUUV can normally be detected in serum from HFRS patients during the first week, and often up to at least 16 days, after onset of symptoms (5–7). However, the development of molecular techniques for PUUV diagnostics has been hindered by the large genomic diversity, even over small geographical distances (7, 9–11).

In this study, all PUUV S segment sequences of Swedish origin available from the GenBank nucleotide collection were used for primer and probe design of a novel PUUV reverse transcription-quantitative (RT)-PCR assay. This assay was then retrospectively evaluated on clinical samples obtained from patients serologically analyzed for HFRS in Sweden from November 2013 to March 2014.

## MATERIALS AND METHODS

### Clinical samples and ethical statement.

HFRS is a notifiable disease in Sweden, and all diagnosed cases are registered in the national HFRS register at the Public Health Agency of Sweden. HFRS laboratory diagnostics are performed at three laboratories in Sweden: the Public Health Agency of Sweden, Umeå University Hospital, and Sunderby Hospital. Serum samples from all patients (*n* = 216) serologically analyzed for HFRS at these three laboratories during November 2013 to March 2014 were collected and stored at −20°C pending analysis. Of these 216 patients, 114 were serologically confirmed for HFRS and reported to the national HFRS register. The remaining 102 individuals whose samples did not show evidence of seroconversion were considered HFRS negative.

RNA preparations from patients with non-HFRS diagnoses (*n* = 47) were tested in the PUUV RT-PCR assay to ensure no unspecific binding of the primers and probe to human RNA. The serum samples were obtained from the biobank repository of the Public Health Agency of Sweden, as stipulated in the regulations for use of such material in diagnostic development and quality assessment. The Swedish Ethical Review Act (2003:16), Ethical Review of Research Involving Humans (http://www.epn.se/media/1205/the_ethical_review_act.pdf), is not applicable for material used in diagnostic development and quality assessment; for this reason, no ethical permit or informed consent was required.

### Assay design.

The assay was designed using all published PUUV S segment sequences (*n* = 44, Swedish origin) covering the complete nucleocapsid protein available in the NCBI GenBank nucleotide collection (see Table A1 in the supplemental material). Multiple sequence alignments were generated using CLC Main Workbench 6.6.5 (CLC Bio, Qiagen). Chemical properties of the primers and probe were evaluated using in-house software and Primer Express v3.0 (Applied Biosystems, Thermo Fisher Scientific).

### Extraction of RNA and real-time RT-PCR.

Total RNA was extracted from 140 μl of serum or from supernatants of infected cells using the QIAamp viral RNA minikit (Qiagen). The RNA was eluted with 60 μl of elution buffer and was stored at −80°C, pending analysis. The PUUV RT-PCR assay was carried out in 25-μl reaction mixtures containing TaqMan Fast Virus 1-step master mix (Applied Biosystems, Thermo Fisher Scientific), 5 μl of template RNA, DNase/RNase-free H_2_O (Life Technologies, Thermo Fisher Scientific), 0.9 μM each primer, and 0.2 μM TaqMan probe (Applied Biosystems). Table 1 shows the characteristics of the primers and probes. Amplification and detection of the 62-bp amplicon were performed in a StepOne Plus real-time PCR system (Applied Biosystems). The cycling profile was as follows: 50°C for 5 min; 95°C for 20 s; and 45 cycles of 95°C for 3 s and 60°C for 30 s. Samples were considered positive if target amplification was recorded within 40 cycles (cycle threshold [*C_T_*], ≤40). The baseline and threshold were set using the autobaseline and autothreshold features in StepOne software v2.2.2 (Applied Biosystems).

**TABLE 1:** Characteristics of primers and probe targeting the S segment of PUUV

Name	Sequence (5′–3′)^*a*^	Position^*b*^	Melting temperature^*c*^ (°C)
PUUV_P	FAM-ACACTGCAAGCAAG-MGB^*d*^	169–182	68.0
PUUV_F	TGGACCCRGATGACGTTAAC	143–162	56.9
PUUV_R1	CAGTGCTGACACTGTYTGTTGC	183–204	58.2
PUUV_R2^*e*^	CAGTGCTGACACTGTCTGTTGT	183–204	55.0

a Degenerated nucleotides: R, A/G; Y, C/T.

b Positions are given according to PUUV strain Umea/hu (GenBank accession no. AY526219).

c The mean melting temperature (*T_m_*) is shown for degenerate primers.

d FAM, 6-carboxy fluorescein; MGB, minor-grove-binding.

e The PUUV_R2 primer is complementary to two sequences (GenBank accession no. GQ339486 and GQ339487) out of the 44 sequences in the data set (see the list of sequences in Technical Appendix 1 in the supplemental material).

To ensure adequate RNA extraction, the presence of β-actin mRNA in clinical samples was analyzed using a TaqMan gene expression assay (catalog no. 4333762F; Applied Biosystems). PUUV RNA was used as the positive control, and water, as negative control.

### Specificity study.

The specificity of the PUUV RT-PCR assay was evaluated by testing RNA extracted from preparations of cell cultures infected with the following viruses: PUUV, strains Bussjö, Kazan, and Sotkamo; other human-pathogenic hantaviruses: Hantaan virus, strain 76-118; Andes virus, strain Chile-9717869; Dobrava virus, strain H119/99; Seoul virus, strain R22; Sin Nombre virus, strain NMR11; Rift Valley fever virus, strain ZH548; Crimean-Congo hemorrhagic fever virus, strain IbAr 10200; Dengue virus 1 through 4, strains 8356/10, 4397/11, 3140/09, and 3274/09, respectively; Japanese encephalitis virus, strain Nakayama; tick-borne encephalitis virus, strain Hochosterwitz; West Nile virus, strain MgAn 786/6/1995; Zika virus, strain MR766; Usutu virus, strain g39; yellow fever virus, strain Asibi; chikungunya virus, strain 23161; and Lassa virus, strain Josiah.

The primer and probe target site in PUUV was tested *in silico* against all non-PUUV sequences in the NCBI nucleotide database by using BLASTn with very loose match criteria (word size, 7; E cutoff, 1,000; match/mismatch cost + 1/−1; Gap cost, 5/2).

### Assay performance.

The amplification efficiency, linear dynamic range, and limit of detection of the PUUV RT-PCR assay were determined by assaying dilutions of *in vitro* transcribed RNA, based on the sequence of PUUV strain Umea/hu (GenBank accession no. AY526219), with the following sequence: 5′-cgtagTGGACCCGGATGACGTTAACAAAAACACACTGCAAGCAAGGCAACAGACAGTGTCAGCACTGtgtca-3′ (BioSynthesis, Inc.).

Possible inhibition of serum, commonly associated with HFRS diagnosis (4-7, 12), and of other matrices, plasma, and saliva (12), was evaluated by comparing the slopes of the standard curves generated by amplification of 5-fold diluted RNA extracted from PUUV (strain Bussjö) spiked in human saliva, serum, and plasma with the slopes for RNA extracted from PUUV diluted in water.

## RESULTS

### Analysis of PUUV sequences and assay design.

**FIG 1:**
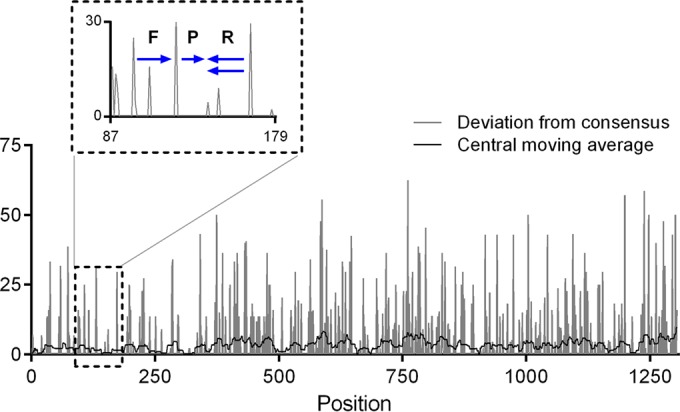
Conservation map of PUUV nucleocapsid protein ORF from Swedish isolates (list of sequences in Table A1 in the supplemental material). Blue arrows represent the position of forward (F) and reverse (R) primers and probe (P) in the PUUV RT-PCR.

Two separate lineages, the North and the South Scandinavian lineages, of PUUV exist in Sweden (9), and our initial analysis showed that there is up to 17% difference in the nucleotide sequence for the nucleocapsid protein open reading frame (ORF) (e.g., Fäboviken/Mg26/05 [GenBank accession no. GQ339484] versus Ljusträsk/Mg20/05 [GenBank accession no. GQ339481]) among the Swedish PUUV isolates. However, a candidate region for primer and probe design with a relatively high sequence conservation was identified in the S segment, where the ORFs of the nonstructural protein S and the nucleocapsid protein overlap. Based on this region, we designed primers and probe for detection of PUUV. Figure 1 shows the location of the primers and probe in relation to the ORF of the nucleocapsid protein and its variability. Table 1 shows the chemical properties of the primers and probe.

### Assay performance.

To determine the linear dynamic range and the amplification efficiency, serial 10-fold dilutions of RNA transcript were tested in triplicates.The linear dynamic range (13) was 10^3^ to 10^10^ genome copy equivalents (GCE)/ml, and the amplification efficiency over that interval was 102% (*R*^2^ = 0.998; *y* intercept = 40).

### Assay sensitivity.

**FIG 2:**
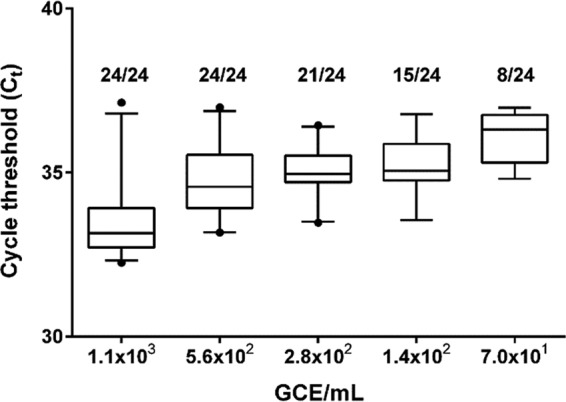
Limit of detection. The number of positives per total number of replicates tested is given above each box. The mean values are indicated by horizontal lines; boxes denote the 25th to 75th percentiles and whiskers, the 5th to 95th percentiles; dots represent outliers. GCE, genome copy equivalents.

To obtain a statistically robust assessment of the limit of detection, RNA transcripts were tested in three parallel experiments each, including eight replicates of RNA copy numbers above and below the expected detection limit. The limit of detection of the PUUV RT-PCR assay was determined to be 560 GCE/ml, representing the lowest RNA copy number for which all 24 replicates of transcript RNA in water were detected (Fig. 2).

Possible PCR inhibition was evaluated by testing virus spiked in clinical matrixes associated with HFRS diagnostics (4-7, 12). The slopes of the lin-log standard curves generated from RNA preparation from saliva, serum, and plasma diluted 5-fold were not significantly different from the slopes obtained from 5-fold dilutions of viral RNA extracted from water (two-way analysis of variance, three replicates: saliva, *P* = 0.45; serum, *P* = 0.63; plasma, *P* = 0.96), indicating that PCR inhibition was minimal in these clinical matrixes.

### Assay specificity.

Cross-reactivity of the PUUV RT-PCR assay with related pathogenic hantaviruses, other members of the Bunyaviridae family, and members of the Arenaviridae, Flaviviridae, and Togaviridae families (the specific viruses tested are listed in Materials and Methods) was excluded by testing RNA preparations from infected cell culture materials. All of these RNA preparations tested negative in the PUUV RT-PCR assay. Further, no relevant hits were obtained when the assay target site was matched against all non-PUUV sequences in the NCBI nucleotide database by using BLASTn (data not shown).

To exclude cross-reactions with human RNA, 47 serum samples from patients with non-HFRS diagnoses were tested using the PUUV RT-PCR assay. None of these samples tested positive in this assay, whereas all were positive for human β-actin mRNA, thus indicating adequate RNA extraction. It was concluded that the PUUV RT-PCR assay could be reliably used for clinical serum samples.

### Comparison of molecular and serological diagnoses, winter 2013 to 2014.

The clinical applicability of the PUUV RT-PCR assay was evaluated by testing serum samples from 216 individuals serologically analyzed for HFRS in Sweden during the winter of 2013 to 2014. Of these, 114 patients were serologically confirmed with HFRS, and 102 were negative for HFRS based on lack of seroconversion.

**FIG 3:**
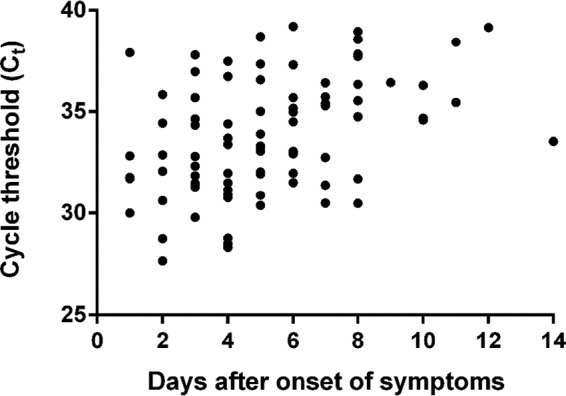
*C_T_* value of positive samples from PUUV-infected patients (*n* = 86) sampled at day 1 to 14 after onset of HFRS. Four patients, sampled at days 5, 9, 10, and 14, were negative by RT-PCR and not included in the graph.

PUUV RNA was detected in 87.7% (*n* = 100) of the 114 HFRS-diagnosed patients, whereas no PUUV RNA was detected in any of the samples from the 102 non-HFRS patients (accuracy, 0.94 [95% confidence interval {CI}, 0.89 to 0.96]; sensitivity, 0.88 [95% CI, 0.80 to 0.93]; specificity, 1 [95% CI, 0.95 to 1]; positive predictive value, 1 [95% CI, 0.95 to 1]; negative predictive value, 0.88 [95% CI, 0.80 to 0.93]). Data regarding days after onset of symptoms was available for 95 of the 114 HFRS-confirmed patients. PUUV RNA were detected in 98.7% (78 of 79; sensitivity, 0.99 [95% CI, 0.93 to 1]) of patients sampled within 8 days after symptomatic onset using the PUUV RT-PCR assay, whereas 56.3% of the patients (9 of 16 patients) sampled at day 9 or later after symptomatic onset tested positive for PUUV RNA. As previously reported (5, 6), the highest levels of PUUV RNA were detected at early time points after the onset of HFRS; a significant correlation (correlation value [*r*], 0.4016; *n* = 86; *P* < 0.001) between *C_T_* values and time after onset of symptoms up to 14 days after infection was observed for samples positive in PUUV RT-PCR (Fig. 3).

### Patients with inconclusive serological results.

For 9.6% (*n* = 11) of the 114 patients with serologically confirmed HFRS, the initial sample arriving to the laboratory tested negative by conventional serologic testing (no virus-specific antibodies were present) or gave inconclusive results. The diagnoses of these patients required additional serological analysis performed on a second sample. In all of these cases, the first sample was positive for PUUV RNA (Table 2), showing that RT-PCR is a sensitive assay for diagnosis of HFRS during the acute phase of disease.

**TABLE 2:** Q-PCR data for patients with first negative or inconclusive serological test

Patient no.	Sex	Age (yrs)	Sample drawn^*a*^		*C_T_* value	
			1st	2nd (days after 1st sample)	1st sample	2nd sample
1	Female	75	NA^*b*^	NA (12)	33.23	38.83
2	Female	25	NA	NA (0.5)	35.46	35.49
3	Male	49	2	11 (9)	32.88	36.12
4	Female	64	6	7 (1)	33.06	33.51
5	Female	49	3	3 (0.5)	31.96	32.81
6	Female	66	4	4 (0.5)	30.69	30.76
7	Female	60	4	5 (1)	32.89	35.02
8	Male	17	7	8 (1)	29.61	30.49
9	Female	39	3	3 (0.5)	34.65	34.56
10	Male	41	3	4 (1)	31.48	32.48
11	Male	67	7	8 (1)	35.30	39.00

a Days after onset of HFRS.

b NA, not available.

## DISCUSSION

Serological assays aimed at detecting acute hantavirus infections can give false-negative, or false-positive, results (14–18). Further, negative or inconclusive results of serological diagnoses are frequently observed during the very early phase of HFRS (2, 5); therefore, analyses of follow-up samples are often required before the diagnosis can be verified. By using molecular methods, a fast and reliable diagnosis of acute virus infection can be obtained. We report here an RT-PCR assay for detection of PUUV RNA that show 98.7% sensitivity within the 8 first days after onset of HFRS and 100% specificity. This RT-PCR assay efficiently detected PUUV in acute HFRS patients from all over Sweden. The results show, to our knowledge for the first time, that RT-PCR can be used for the routine diagnosis of HFRS.

Rapid and reliable diagnosis of hantavirus infections is of importance to initiate supportive care in severe cases, avoid unnecessary examinations and antibiotic treatment, and initiate preventive measures to avoid exposure to others (19–21). By serological diagnosis, 11 patients could not be diagnosed until a second sample was tested. In contrast, PUUV RNA was detected in all of the first samples, and, in these cases (representing 9.6% of all 114 HFRS patients in this study), a faster diagnosis could have been obtained by using molecular diagnosis.

Hantaviruses are negative-stranded RNA viruses with tripartite genomes encoding 4 or 5 proteins (1, 22). All HPS-causing hantaviruses and PUUV, but no other HFRS-causing hantaviruses, have a short ORF within the nucleocapsid protein ORF that encodes the nonstructural protein NSs. This highly conserved region of the S segment, corresponding to the overlapping ORFs of the nucleocapsid protein and the NSs proteins, might be an attractive target for molecular diagnostics of HPS-causing hantaviruses.

The results from this study suggest that the PUUV RT-PCR assay can be complementary, or even an alternative, to serological assays in the diagnosis of PUUV-caused HFRS.

## Supplementary Material

Supplemental material
