# Supplementary material for: Molecular Diagnosis of Hemorrhagic Fever with Renal Syndrome Caused by Puumala Virus
Source: J Clin Microbiol. 2016 Apr 25;54(5):1335–9. doi: 10.1128/JCM.00113-16 (PMC4844727; doi:10.1128/JCM.00113-16)
Supplement: Supplemental material [file supp_54_5_1335__index.html]

Supplemental material 

# Molecular Diagnosis of Hemorrhagic Fever with Renal Syndrome Caused by Puumala Virus

## Supplemental material

- Supplemental file 1 -

  Table SA1 (GenBank accession numbers for PUUV sequences used in the design of the assay)

  PDF, 7.1K
