# Supplementary material for: Molecular Diagnosis of Hemorrhagic Fever with Renal Syndrome Caused by Puumala Virus
Source: J Clin Microbiol. 2016 Apr 25;54(5):1335–9. doi: 10.1128/JCM.00113-16 (PMC4844727; doi:10.1128/JCM.00113-16)
Supplement: Supplemental material [file JCM.00113-16_zjm999094947so1.pdf]

Appendixes

Table A1. Sequences, Swedish PUUV-isolates.

AM746327_Pålböle

AM746321_Norum

AM746323_Norum

AM746320_Norum

AM746322_Norum

AM746326_Norum

AM746313_Djäkneböle

AM746311_Djäkneböle

AM746312_Djäkneböle

AM746310_Djäkneböle

AM746314_Djäkneböle

U141137_Hällnäs

AJ223380_Tavelsjö

AJ223371_Huggberget

Z48586_Vindeln

AJ223374_Mellansel

AJ223375_Mellansel

AY526219_Bussjö

AM746331_Skäran

AM746332_Skäran

AM746333_Skäran

AM746319_Gumboda

AM746317_Gumboda

AM746315_Gumboda

AM746316_Gumboda

AM746318_Gumboda

AM746330_Pålböle

AM746325_Norum

AM746324_Norum

AM746328_Pålböle

AM746329_Pålböle

GQ339476_Aijäjärvi

GQ339477_Aijäjärvi

GQ339482_Kälvudden

GQ339478_Jockfall

GQ339480_Gyttjeå

GQ339479_Muskosel

GQ339481_Ljusträsk

GQ339483_Bergsjöbo

AJ223377_Sollefteå

GQ339484_Fäbodviken

AJ223376_Sollefteå

GQ339486_Munga

GQ339487_Munga
